# Chemical Cleaning of Ultrafiltration Membrane Fouled by Humic Substances: Comparison between Hydrogen Peroxide and Sodium Hypochlorite

**DOI:** 10.3390/ijerph16142568

**Published:** 2019-07-18

**Authors:** Kai Li, Shu Li, Tinglin Huang, Chongzhe Dong, Jiawei Li, Bo Zhao, Shujia Zhang

**Affiliations:** 1 Key Laboratory of Northwest Water Resource, Environment and Ecology, MOE, Xi’an University of Architecture and Technology, Xi’an 710055, China; 2 Shaanxi Key Laboratory of Environmental Engineering, Xi’an University of Architecture and Technology, Xi’an 710055, China

**Keywords:** ultrafiltration (UF) membrane, humic substances (HS) fouling, chemical cleaning, hydrogen peroxide (H_2_O_2_), sodium hypochlorite (NaClO)

## Abstract

Chemical cleaning is indispensable for the sustainable operation of ultrafiltration (UF) system in water and wastewater treatment. Sodium hypochlorite (NaClO) is an established cleaning agent for membranes subject to organic and microbial fouling, but concerns have been raised about the generation of toxic halogenated by-products during NaClO cleaning. Hydrogen peroxide (H_2_O_2_) is a potential “green” cleaning agent that can avoid the formation of halogenated by-products. In this work, cleaning efficacy of H_2_O_2_ and NaClO for UF membrane fouled by humic substances (HS) was evaluated under a wide pH range, and change of HS’s properties due to reaction with cleaning agents was examined. The cleaning efficacy of H_2_O_2_ was lower than that of NaClO at pH 3–9, but it increased to a level (91.4%) comparable with that of NaClO at pH 11. The extents of changes in properties and fouling potential of HS due to reacting with cleaning agents were consistent with their cleaning efficacy. H_2_O_2_ treatment at pH 11 significantly increased negative charge of HS molecules, decomposed high-MW molecules, and reduced its fouling potential. Therefore, considering treatment/disposal of cleaning waste and cleaning efficacy, H_2_O_2_ cleaning under strong alkaline condition can be a good choice for HS-fouled membrane.

## 1. Introduction

In the past few decades, the application of ultrafiltration (UF) in water industry has increased rapidly because of its small footprint and efficient and reliable removal towards particles and pathogens [1,2]. Nevertheless, membrane fouling, i.e., the decrease of membrane permeability due to accumulation of organic/inorganic/biological substances on/within membrane, is one of the major bottlenecks of UF technology [3,4]. Membrane fouling results in the decline of membrane flux for constant pressure system or the increase of trans-membrane pressure (TMP) for constant flux system, which would impair system productivity and increase operating costs [5]. Although many efforts, such as pretreatment of feed water [6,7], membrane material modification [8,9], and operation optimization [10,11], have been made to mitigate membrane fouling, build-up of physically irreversible fouling during long-term operation is still inevitable [12]. Therefore, chemical cleaning has to be conducted periodically to remove foulants deposited on/with membrane and restore membrane permeability [13,14].

Many chemical reagents have been used as cleaning agents for UF membrane, including acids, alkalis, oxidants, surfactants, and complexing agents [15]. Among them, sodium hypochlorite (NaClO) is one of the most commonly used oxidative cleaning agents because of its cost-effectiveness and ease to use. For organic fouling, NaClO cleaning can alter functional groups, molecular size, surface charge, and hydrophilicity of organics, resulting in the decrease of foulants-membrane interactions and dislodgement of organic foulants [12,16,17]. However, the reactions between NaClO and organic foulants/membrane material would inevitably generate toxic halogenated by-products [18,19,20,21]. It is estimated that the total organic halogenated material discharged by on-line NaClO cleaning of membrane bioreactors (MBRs) in China would reach 648.45 kg/year according to the current MBR processing capacity and cleaning conditions [22]. Therefore, NaClO cleaning waste should be carefully treated and disposed, leading to the increase of chemicals consumption and operation costs.

Compared with NaClO, hydrogen peroxide (H_2_O_2_) is regarded as a green oxidant because its reduction product is water (H_2_O), and the formation of halogenated by-products can be avoided. Although reactivity of H_2_O_2_ is restricted by a high activation energy barrier, it is still a potential oxidative cleaning agent and has been investigated in several studies. For polysulfone membrane fouled by fermentation broth, flux was restored to 80% of the initial flux of new membrane after washing with 3 g/L of H_2_O_2_ (pH = 3.6), which was significantly higher than HCl and NaOH [23]. Strugholtz et al. [24] examined cleaning efficacy of H_2_O_2_ for membrane fouled by flocculated reservoir water, and no flux recovery was obtained with no pH adjustment, but the cleaning efficacy was significantly improved after adjusting solution pH to 12. Wang et al. [25] reported that H_2_O_2_ cleaning obtained the highest flux recovery for fouled forward osmosis membrane in anaerobic osmotic MBR, whereas chelate, surfactant, acids, and alkali cannot effectively remove foulants on the membrane. However, Kuzmenko et al. [26] found that H_2_O_2_ cleaning (pH and concentration not given) even reduced membrane flux further (the specific flux decreased from 0.60 to 0.52), whereas NaClO cleaning achieved significant flux recovery. For polyethersulfone (PES) membrane fouled by paper-making wastewater, no flux recovery was achieved after cleaning with 2% H_2_O_2_ (without pH adjustment) [27]. In general, compared with NaClO cleaning, much less attention has been paid to H_2_O_2_ cleaning, and the cleaning efficacy reported in literature seems contradictory. Meanwhile, cleaning mechanisms of H_2_O_2_ and its interactions with organic membrane foulants are still unclear.

H_2_O_2_ is a very weak acid with a pKa of 11.62 (T = 25 °C), and only under alkaline conditions it would be dissociated to form HO_2_^−^, which is believed to be an active species for bleaching and degradation of some dyes. Moreover, several studies reported that H_2_O_2_ can be activated by alkali to generate reactive oxygen species, such as superoxide radical (O_2_^−^) and singlet oxygen (^1^O_2_) [28]. As a result, it is expected that the cleaning efficacy of H_2_O_2_ would be strongly affected by solution pH, but few studies have comprehensively examined this issue. In this work, cleaning efficacy of H_2_O_2_ for UF membrane fouled by humic substance (HS) at a wide pH range (3–11) was investigated, and NaClO cleaning was conducted as the reference. To elucidate cleaning mechanisms, fouling potential and properties of HS before and after reaction with cleaning agents at optimum pH were analyzed.

## 2. Materials and Methods

### 2.1. Membrane and Filtration Set-Up

Flat-sheet PES membranes (UP150, Microdyn-Nadir, Wiesbaden, Germany) with a molecular weight cutoff (MWCO) of 150 kDa and an effective surface area of 45 cm^2^ were used for evaluation of both cleaning efficacy and fouling potential of HS. According to the manufacturer, the membrane material is blended with hydrophilic additives, and pure water contact angle of the membrane is 45 ± 4 °C. Meanwhile, the membrane surface is negatively charged with a zeta potential of −17 ± 3 mV at pH 7. To ensure thorough removal of preservative agents, new membranes were soaked in ultrapure water (18.2 MΩ cm, ELGA LabWater’s, High Wycombe, UK) for at least 24 h, and 150 mL ultrapure water was filtered before use. The initial pure water flux of the membranes used in this study was in the range of 410 ± 10 L/(m^2^·h) at a TMP of 100 kPa.

Filtration experiments were carried out in a filtration cell (Amicon 8400, Millipore, Burlington, MA, USA) in dead-end mode at room temperature (25 ± 1 °C). During filtration, the glossy side of the membrane faced the feed solution, and the membrane can be backwashed by placing the reverse side of the membrane upwards. Nitrogen gas was used to drive feed solution through the membrane, and a constant pressure of 100 kPa was used in this experiment. Permeate was collected into a conical flask placed on an electronic balance connected to a computer, and the weight data were automatically recorded every 5 s.

### 2.2. Preparation of HS-Fouled Membranes

To evaluate the cleaning efficacy of H_2_O_2_ and NaClO at various pH, PES membranes fouled by HS to a similar extent were prepared by filtering HS solution using the membrane and filtration set-up described in Section 2.1. Humic acid obtained from Sigma-Aldrich Chemical Co. (St. Louis, MO, USA) was used as the representative HS. To speed up membrane fouling, a relatively high concentration of HS (i.e., 50 mg/L) was used and the corresponding dissolved organic carbon (DOC) concentration was 20.5 ± 0.6 mg/L. Meanwhile, 1 mmol/L CaCl_2_, 1 mmol/L NaHCO_3_, and 6 mmol/L NaCl were added to simulate the solution chemistry in natural water. To focus on physically irreversible fouling, the membrane was backwashed with 50 mL ultrapure water after filtering 350 mL HS solution. Based on preliminary experiments (data not shown), two cycles of filtration–backwash were required to obtain a HS-fouled membrane with flux decreasing to 10–15% of the initial value. The flux of new membrane and fouled membrane was denoted as J_0_ and J_f_, respectively.

### 2.3. Cleaning Process and Cleaning Efficacy Evaluation

All chemicals and reagents used in this study were in analytical grade. Commercially available NaClO (~10 % in weight) and H_2_O_2_ (~30 % in weight) were purchased from Tianli Chemical Reagent Co. (Tianjin, China) and Kermel Chemical Reagent Co. (Tianjin, China), respectively. Concentrations of NaClO and H_2_O_2_ solution were determined by iodometric titration method and permanganate titration method, respectively, and therefore the reported concentrations were sum of all active species in the solutions. NaClO and H_2_O_2_ cleaning solutions were both diluted to 500 mg/L using ultrapure water and their pH were adjusted to 3, 5, 7, 9, and 11 with HCl or NaOH. HCl and NaOH were both obtained from Kermel Chemical Reagent Co. (Tianjin, China). Meanwhile, pure water at the pH of 3, 5, 7, 9, and 11 was also used as control cleaning solution.

HS-fouled membranes were taken out of the filtration cell and immersed in glassware containing 50 mL corresponding cleaning solutions. After 6 h of static soaking, the membranes were rinsed with pure water to remove cleaning agents, and their pure water flux was determined and denoted as J_c_. Each cleaning test was conducted in triplicate.

Therefore, the cleaning efficacy of different cleaning solutions can be quantified based on flux recovery ratio (Equation (1)).

(1) $$\mathrm{Cleaning}\;\mathrm{efficacy}\;\left(\%\right)=\frac{J_{c}-J_{f}}{J_{0}-J_{f}}\times 100\%$$

### 2.4. Reaction of HS with Cleaning Agents

To elucidate cleaning mechanisms of NaClO and H_2_O_2_ towards HS fouling, effects of reaction with cleaning agents on fouling potential and properties of HS were examined. HS solutions (100 mg/L) at certain pH were dosed with predetermined NaClO or H_2_O_2_ to obtain an oxidant/DOC ratio of 13:1. Only the pH of 9 and 11 were investigated because H_2_O_2_ exhibited significant different cleaning efficacy under these two pH, while NaClO cleaning was commonly conducted under alkaline conditions. After 6 h of reaction, HS solutions were taken out to perform filtration test and determination of UV-Vis absorbance spectra, zeta potential and MW distribution. Meanwhile, HS solutions at the same concentration and pH were denoted as raw HS solutions.

### 2.5. Characterization of Fouling Potential and Properties of HS before and after Reacting with Cleaning Agents

Fouling potential of raw and cleaning agent-treated HS was evaluated by single-cycle filtration test with the membrane and filtration cell described in Section 2.1. Raw and cleaning agent-treated HS solutions were diluted by 10 times before filtration test, and the initial volume of feed solution was 350 mL. When the permeate volume reached 300 mL, the concentrate with a volume of 50 mL was discarded, and the membrane was backwashed with 50 mL pure water. Therefore, the final volume reduction ratio was 7, and the cumulative volume of permeate per unit membrane surface area (V_s_) was 0.067 m^3^/m^2^. Unified membrane fouling index (UMFI) can be used to assess membrane fouling quantitatively [29]. Based on the permeate flux of new membrane (J_0_), the final flux at the end of filtration (J_1_) and the flux after backwash (J_2_), total fouling index, (TFI) and hydraulically irreversible fouling index (HIFI) can be calculated according to Equations (2) and (3), respectively. All filtration tests were conducted in triplicate.

(2) $$\mathrm{TFI}\;\left(m^{-1}\right)=\frac{J_{0}/J_{1}-1}{V_{s}}$$

(3) $$\mathrm{HIFI}\;\left(m^{-1}\right)=\frac{J_{0}/J_{2}-1}{V_{s}}$$

UV-Vis absorbance spectra of HS solution were measured using U-3900 UV/vis spectrophotometer (Hitachi Ltd, Tokyo, Japan). Differential log transformed absorbance spectra (*DLnA*) and the spectral parameter (*DSlope*_325–375_) were calculated according to literature and were used as indication of HS properties [30].

Zeta potential of HS was determined by using Nano S90 (Malvern Panalytical Ltd, Malvern, UK). DOC was measured by a total organic carbon analyzer (multi N/C2100, Analytik Jena AG, Jena, Germany). All measurements were done in triplicate.

Molecular weight (MW) distributions of raw and cleaning agent-treated HS were determined using UF separation method in parallel mode. Regenerated cellulose membranes with MWCO of 100, 30, 10 kDa (Amicon YM 100, YM 30, YM 10, Millipore, Burlington, MA, USA) were used. All MW distribution tests were conducted in duplicate, and the detailed procedure of the test can be found in previous literature [31].

## 3. Results and Discussion

### 3.1. Efficacy of H_2_O_2_ and NaClO Cleaning under Various pH

**Figure 1:**
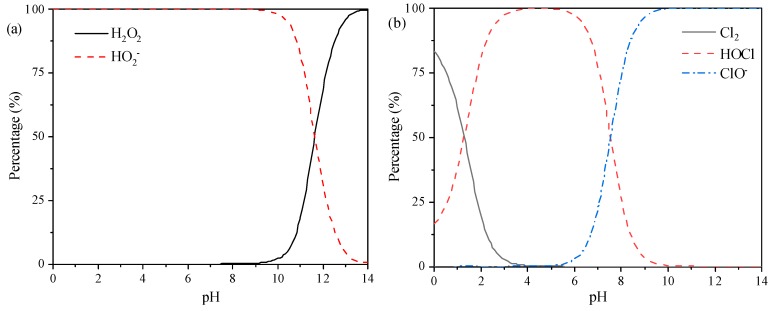
Distribution of main species in H_2_O_2_ (**a**) and NaClO (**b**) solutions as a function of pH at 25 °C and for *C*(H_2_O_2_)_T_ = *C*(NaClO)_T_ = 500 mg/L.

For both H_2_O_2_ and NaClO solutions, there is an equilibrium between two or more species depending on solution pH and temperature. H_2_O_2_ is a weak acid that dissociates in aqueous solution according to Equation (4) [32], while three chlorine species, i.e., Cl_2_, HOCl, and ClO^–^, coexist in NaClO solution (Equations (5) and (6)) [33]. For 500 mg/L H_2_O_2_ and NaClO solution, the distribution of main species as a function of pH at 25 °C is shown in Figure 1a,b, respectively.

(4) $$H_{2}O_{2}⇌{\mathrm{HO}}_{2}{}^{-}+H^{+}\;{\;k}_{1}=2.2\times 10^{-12}\;\left(T=25\;{}^{\circ}C\right)$$

(5) $${\mathrm{Cl}}_{2}+H_{2}O⇌\mathrm{HOCl}+{\mathrm{Cl}}^{-}+H^{+}\;{\;k}_{2}=5.1\times 10^{-4}\;\left(T=25\;{}^{\circ}C\right)$$

(6) $$\mathrm{HOCl}⇌{\mathrm{ClO}}^{-}+H^{+}\;{\;k}_{3}=2.9\times 10^{-8}\;\left(T=25\;{}^{\circ}C\right)$$

As for H_2_O_2_ solution, almost no dissociation of H_2_O_2_ occurs at pH < 9, while the percentage of HO_2_^–^ increases rapidly when the solution pH exceeds 10. At the pH of 11, about 18% of H_2_O_2_ is dissociated to the form of HO_2_^–^. Based on the standard electrode potential (E_0_), oxidation capacity of H_2_O_2_/H_2_O (*E_0_* = 1.78 V) is stronger than HO_2_^–^/OH^–^ (*E_0_* = 0.88 V). With respect to NaClO solution, Cl_2_ and HOCl are the dominant species at pH < 4, while HOCl and ClO^–^ coexist at pH 5–10. At pH > 10, almost all chlorine exists in the form of ClO^–^. The oxidation capacity of chlorine species is in the range of HClO/Cl^–^ (*E_0_* = 1.49 V) > Cl_2_ (aq)/Cl^–^ (*E_0_* = 1.40 V) > ClO^–^/Cl^–^ (*E_0_* = 0.89 V). As a result, it can be speculated that the oxidation capacity of both H_2_O_2_ and NaClO solutions would decrease with the increase of pH.

**Figure 2:**
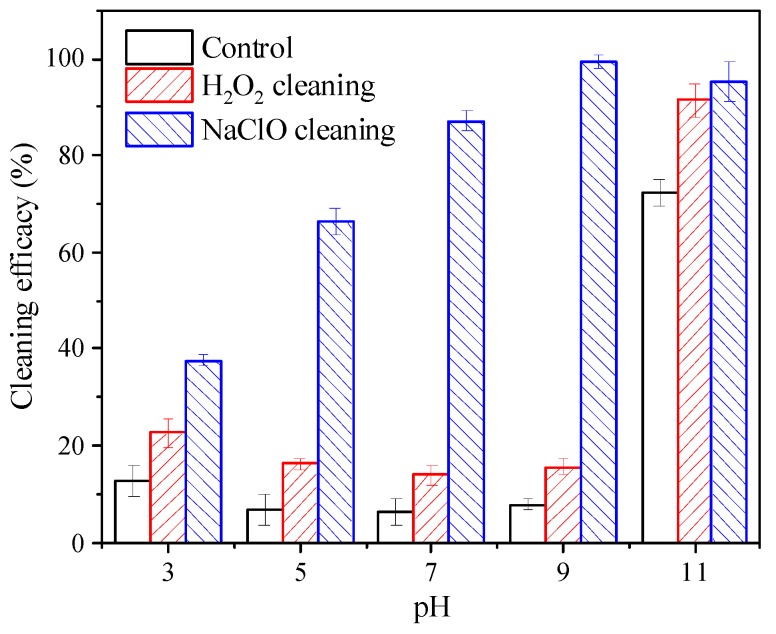
Effects of pH on cleaning efficacy of H_2_O_2_ and NaClO for ultrafiltration (UF) membranes fouled by humic substances (HS). *C*(H_2_O_2_)_T_ = *C*(NaClO)_T_ = 500 mg/L, with pure water as control, and pH was adjusted by adding HCl or NaOH; cleaning time 6 h.

Effects of pH on cleaning efficacy of H_2_O_2_ and NaClO for HS-fouled membrane are shown in Figure 2. For the control group, alkaline cleaning at pH 11 achieved the highest cleaning efficacy (72.2%), whereas acid cleaning was just slightly better than cleaning by pure water. The major mechanism of alkaline cleaning for organic fouling was solubilization and hydrolysis of organic foulants, which promotes swelling of the fouling layer and detachment from membrane surface [24,34,35]. As for H_2_O_2_, the cleaning efficacy at pH 3 was 22.6%, and it decreased slightly to 13.9–16.3% at pH 5–9. The results seemed to be consistent with the decrease of oxidation capacity, but the cleaning efficacy at pH 11 increased remarkably to 91.4%. Even taking into account the cleaning efficacy of alkaline cleaning, the contribution of H_2_O_2_ was higher at pH 11. Strugholtz et al. [24] also reported the increase of H_2_O_2_ cleaning efficacy due to combination with NaOH, but the reason was not explored. With respect to NaClO, the cleaning efficacy improved with the increase of pH from 3 to 9, and the cleaning efficacy was 99.4% and 95.2% at pH 9 and 11, respectively. Wang et al. [36] observed similar results and ascribed the better performance at higher pH to the uneven and fast diffusion of ClO^−^, but the variation of properties of organic foulants during chemical cleaning was not examined. As shown in Figure S1 in the Supplementary Materials, PES membrane was stable after exposure to these cleaning agents at pH 11, suggesting the recovery of permeability was not due to membrane damage. In short, for HS-fouled UF membrane, H_2_O_2_ cleaning at pH 11 might be a feasible alternative for NaClO cleaning considering cleaning efficacy and reduction of chlorinated by-products.

### 3.2. Fouling Potential of HS before and after Reacting with H_2_O_2_ and NaClO

**Figure 3:**
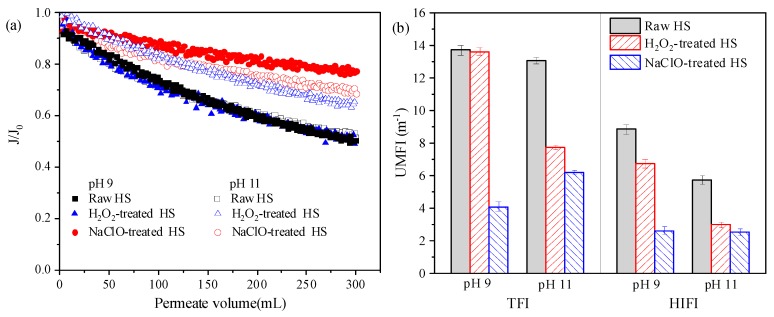
Fouling potential of HS before and after reacting with H_2_O_2_ and NaClO: (**a**) Flux decline, (**b**) unified membrane fouling index (UMFI). The ratio of oxidant to dissolved organic carbon (DOC): 13:1; reaction time: 6 h. (TFI: total fouling index, HIFI: hydraulically irreversible fouling index).

To verify the effectiveness of H_2_O_2_ in membrane cleaning, fouling potential of HS before and after reacting with H_2_O_2_ and NaClO at pH 9 and 11 was investigated, and the results are shown in Figure 3. It can be seen that raw HS solutions at pH 9 and 11 resulted in similar flux decline pattern, and permeate flux at the end of filtration cycle decreased to about 51% of the initial flux. The trend was not affected by H_2_O_2_ treatment at pH 9, whereas flux decline was significantly alleviated due to H_2_O_2_ treatment at pH 11, with the ending flux accounting for 65% of the initial one. Flux decline was substantially abated by NaClO treatment at both pH 9 and 11, and the final flux was 77% and 68% of the initial flux, respectively. As shown in Figure S2 in the Supplementary Materials, rejection of HS was significantly reduced due to reacting with H_2_O_2_ at pH 11 and with NaClO at pH 9 and 11, which was consistent with the flux decline trends. Hydraulically irreversible fouling was quantified by HIFI and the results are presented in Figure 3b. At pH 9, much lower irreversible fouling occurred after NaClO treatment, with H_2_O_2_ and NaClO treatment reducing HIFI by 24.0% and 70.5%, respectively. For pH 11, H_2_O_2_ and NaClO treatment resulted in 48.4% and 56.4% decrease of HIFI, respectively.

In short, H_2_O_2_ treatment at pH 9 exerted minor influence on fouling potential of HS, whereas H_2_O_2_ treatment at pH 11 remarkably decreased both total and irreversible fouling caused by HS. As for NaClO treatment, fouling potential of HS was effectively reduced at both pH 9 and 11, and the decrease of total fouling was a little more pronounced at pH 9. These results were consistent with the cleaning efficacy of H_2_O_2_ and NaClO, as illustrated in Figure 2. At pH 11, both H_2_O_2_ and NaClO treatment significantly decreased fouling potential of HS, indicating that HS properties were obviously changed by these two cleaning agents. Therefore, the significant increase of H_2_O_2_ cleaning efficacy with the increase of pH from 9 to 11 should not be solely attributed to alkali solubilization, and variation of HS properties played an important role.

### 3.3. Alteration of HS Properties Due to Reacting with H_2_O_2_ and NaClO

To elucidate cleaning mechanisms of H_2_O_2_ and NaClO at pH 9 and 11, several properties of HS before and after reacting with H_2_O_2_ and NaClO were examined. It should be noted that the decrease of DOC caused by reacting with H_2_O_2_ and NaClO was less than 10%, suggesting minimal mineralization of HS during reactions.

**Figure 4:**
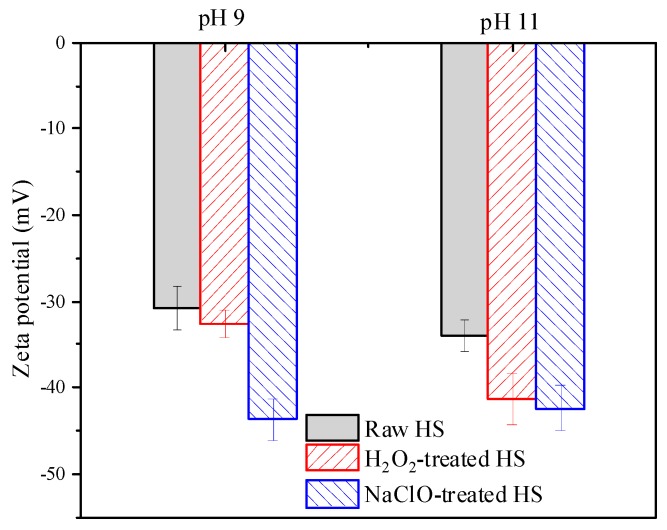
Zeta potential of HS before and after reacting with H_2_O_2_ and NaClO. The ratio of oxidant to DOC: 13:1; reaction time: 6 h.

Figure 4 presents zeta potential of HS before and after reacting with H_2_O_2_ and NaClO. It can be seen that HS was negatively charged, and zeta potential of raw HS at pH 9 and 11 was −30.8 and −34.1 mV, respectively. At pH 9, zeta potential of HS was only slightly decreased by H_2_O_2_ treatment, whereas it was obviously decreased to −43.7 mV by NaClO treatment. For pH 11, zeta potential of HS was decreased to −41.3 and −42.4 mV due to H_2_O_2_ and NaClO treatment, respectively. Because the membrane used in this study was negatively charged (−15.9 ± 0.3 mV in 1 mM KCl solution at pH 7), the decrease of zeta potential, i.e., the increase of negative charge, would enhance electrostatic repulsion and weaken the adhesion force between HS and the membrane [2]. Therefore, the higher cleaning efficacy of H_2_O_2_ at pH 11 can be partly attributed to the more significant decrease of zeta potential caused by H_2_O_2_ treatment at pH 11.

**Figure 5:**
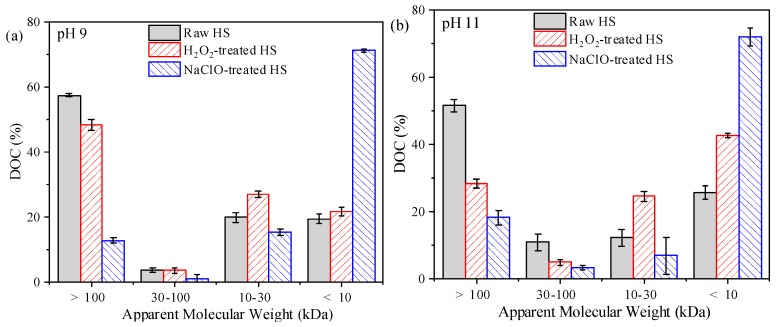
Apparent molecular weight distributions of HS before and after reacting with H_2_O_2_ and NaClO: (**a**) pH 9, (**b**) pH 11. The ratio of oxidant to DOC: 13:1; reaction time: 6 h.

MW distributions of HS before and after reacting with H_2_O_2_ and NaClO are shown in Figure 5. At pH 9, the fraction of high-MW (>100 kDa) accounted for 57.3% of raw HS based on DOC, while the fractions with MW of 10–30 kDa and <10 kDa made up 19.8% and 19.3%, respectively. After H_2_O_2_ treatment, the ratio of the high-MW fraction slightly decreased to 48.2%, accompanying with some increase of the 10–30 kDa fraction. In contrast, NaClO treatment led to substantial decrease of the high-MW fraction, and the percentage of the fraction with MW of <10 kDa was increased remarkably to 71.2%. At pH 11, raw HS exhibited similar MW distribution with that at pH 9, but the change caused by H_2_O_2_ treatment was much more obvious. After H_2_O_2_ treatment, proportion of the high-MW fraction was decreased from 51.5% to 28.2%, while ratios of the fractions with MW of 10–30 kDa and < 10 kDa were increased to 24.4% and 42.6%, respectively.

**Figure 6:**
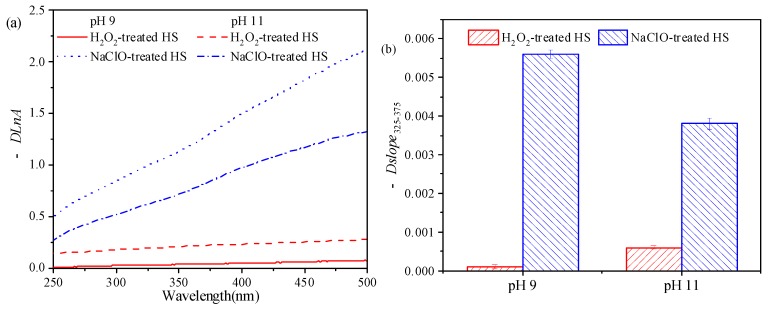
Differential log-transformed absorbance spectra (−*DLnA*) (**a**) and change in spectral parameter (−*DSlope*_325–375_) (**b**) of HS before and after reacting with H_2_O_2_ and NaClO. The ratio of oxidant to DOC: 13:1; reaction time: 6 h.

UV-Vis spectral parameters can provide abundant information about the composition and structure of HS and have been successfully applied to characterize properties of HS [37], binding of metal ions on it [38,39], as well as its reactions with oxidants [40]. The differential log-transformed absorbance spectra (*DLnA*) and variation in spectral slope determined in the wavelength range from 325 to 375 nm (*DSlope*_325–375_) of HS before and after reacting with H_2_O_2_ and NaClO are shown in Figure 6. It can be seen that the absorbance spectra of HS changed greatly after NaClO treatment, and the change at pH 9 was more remarkable than that at pH 11. In comparison, variations of the absorbance spectra due to H_2_O_2_ treatment were insignificant. Considering the significant change of zeta potential and MW distribution of HS due to H_2_O_2_ treatment at pH 11, it seems that UV-Vis spectra was not suitable for the characterization of the reaction between HS and H_2_O_2_.

Based on the alteration of zeta potential, MW distribution, and absorbance spectra parameters of HS before and after reacting with two cleaning agents, it can be concluded that NaClO can effectively oxidize HS at both pH 9 and 11, while HS can only be oxidized by H_2_O_2_ at pH 11. The results were consistent with their cleaning efficacy and the corresponding fouling potential. For NaClO, the greater change of HS properties at pH 9 can be attributed to the higher *E*_0_ of HClO species and generation of OH due to the coexistence of HClO and ClO^−^ [41]. With respect to H_2_O_2_, the discrepancy of oxidation capacity at pH 9 and 11 cannot be explained by the *E*_0_ of H_2_O_2_ (1.78 V) and HO_2_^−^ (0.88 V). The generation of various reactive oxygen species in H_2_O_2_ solution under strong alkaline condition might be responsible for the higher cleaning efficacy and oxidation capacity of H_2_O_2_ towards HS at pH 11 [28,32].

## 4. Conclusions

In this study, cleaning efficacy of H_2_O_2_ and NaClO at a wide pH range (3–11) for UF membrane fouled by HS was investigated, and properties of HS before and after reacting with cleaning agents were analyzed. The cleaning efficacy of H_2_O_2_ was lower than that of NaClO at pH 3–9, while it increased significantly to 91.4% and was comparable with that of NaClO at pH 11. The extents of changes in properties and fouling potential of HS due to reacting with H_2_O_2_ and NaClO at both pH 9 and 11 was consistent with the cleaning efficacy. H_2_O_2_ treatment exerted minor influence on HS properties at pH 9, but it led to significant increase of negative charge, decomposition of high-MW molecules, and reduction of both total and irreversible fouling at pH 11. Considering the cleaning efficacy and control of chlorinated by-products during chemical cleaning, H_2_O_2_ cleaning under strong alkaline condition can be a good alternative for NaClO cleaning for HS-fouled UF membrane.

## Supplementary Materials

The following are available online at https://www.mdpi.com/1660-4601/16/14/2568/s1, Figure S1: SEM images of pristine membrane and membranes exposure to cleaning agents at pH 11 for 6 h (× 100,000 magnification): (a) pristine membrane, (b) pure water, (c) 500 mg/L H_2_O_2_, (d) 500 mg/L NaClO; Figure S2: Rejection of HS before and after reacting with H_2_O_2_ and NaClO. The ratio of oxidant to DOC: 13:1; reaction time: 6 h.
