# Supplementary material for: Chemical Cleaning of Ultrafiltration Membrane Fouled by Humic Substances: Comparison between Hydrogen Peroxide and Sodium Hypochlorite
Source: Int J Environ Res Public Health. 2019 Jul 18;16(14):2568. doi: 10.3390/ijerph16142568 (PMC6678075; doi:10.3390/ijerph16142568)
Supplement: Supplementary file 1 [file ijerph-16-02568-s001.pdf]

Supplementary Information

Chemical Cleaning of Ultrafiltration Membrane Fouled by Humic Substances: Comparison between Hydrogen Peroxide and Sodium Hypochlorite

Kai Li [1,2,*], Shu Li [1,2], Tinglin Huang [1,2,*], Chongzhe Dong [1,2], Jiawei Li [1,2], Bo Zhao [1,2] and Shujia Zhang [1,2]

[1] Key Laboratory of Northwest Water Resource, Environment and Ecology, MOE, Xi'an University of Architecture and Technology, Xi'an 710055, China; sisl2018@163.com (S.L.); dongchongzhe97@163.com (C.D.); l13072990283@163.com (J.L.); 18709297271@163.com (B.Z.); zhangshujia@xauat.edu.cn (S.Z.)

[2] Shaanxi Key Laboratory of Environmental Engineering, Xi'an University of Architecture and Technology, Xi'an 710055, China

[*] Correspondence: likai@xauat.edu.cn (K.L.); huangtinglin@xauat.edu.cn (T.H.); Tel.: +86-029-8220-7886 (K.L.); +86-029-8220-1038 (T.H.)

To verify the stability of the PES membrane upon chemical cleaning, scanning electron microscopy (SEM, FEI Quanta 600) was used to characterize the morphology of pristine membrane and membranes exposure to pure water, 500 mg/L H_2O_2, 500 mg/L NaClO at pH 11 for 6 h were taken and shown in Figure S1. It can be seen that there was no obvious difference in the morphology of these membranes, suggesting that membrane stability was not compromised by these cleaning reagents at the exposure intensity of 3 g·h/L.

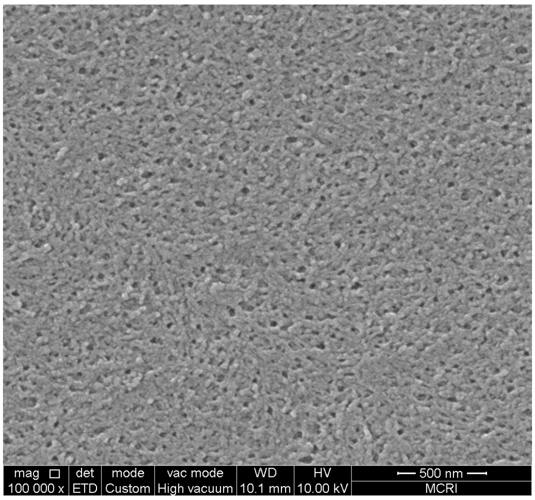

(**a**)

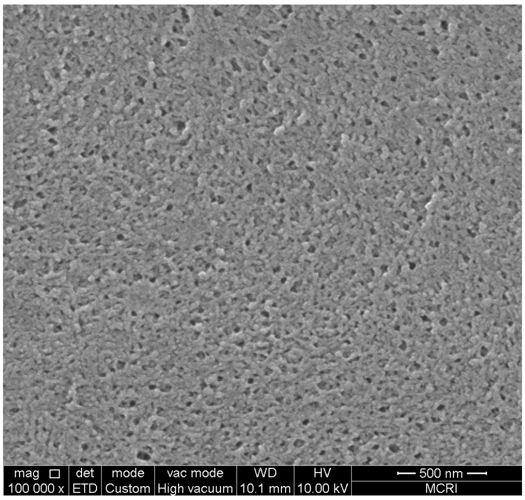

(**b**)

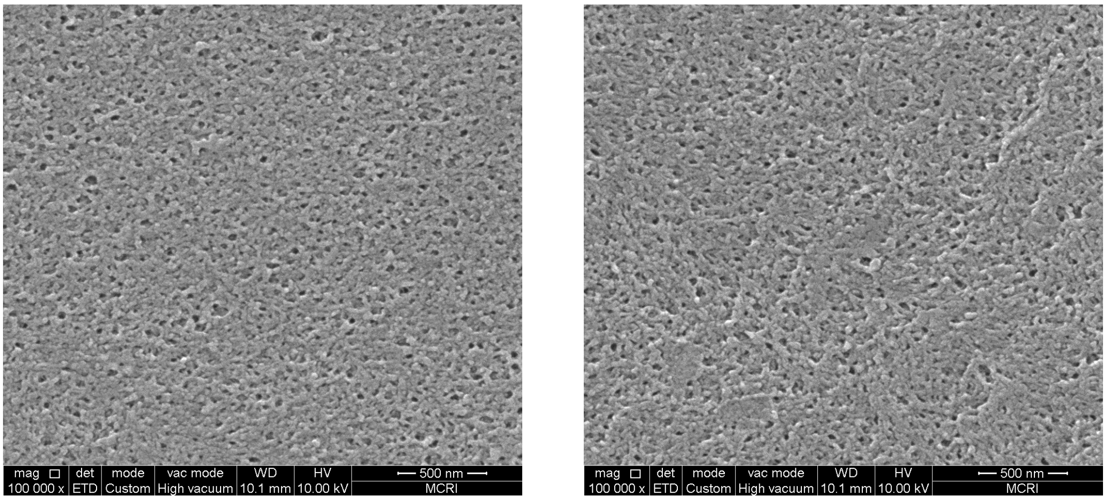

(**c**) (**d**)

Figure S1. SEM images of pristine membrane and membranes exposure to cleaning agents at pH 11 for 6 h (× 100,000 magnification): (**a**) pristine membrane, (**b**) pure water, (**c**) 500 mg/L H_2O_2, (**d**) 500 mg/L NaClO.

Rejection of HS before and after reacting with H_2O_2 and NaClO by PES membrane are shown in Figure S2. It can be seen that about 55% and 50% raw HS was rejected by the PES membrane at pH 9 and 11, respectively. The result is consistent with previous reports [1–3]. The rejection rate was a little lower at pH 11 because its solubility increased at higher pH. HS rejection was not affected by H_2O_2 treatment at pH 9, whereas it decreased significantly to 23% due to H_2O_2 treatment at pH 11. After NaClO treatment at pH 9 and 11, HS rejection substantially decreased to 12% and 20%, respectively.

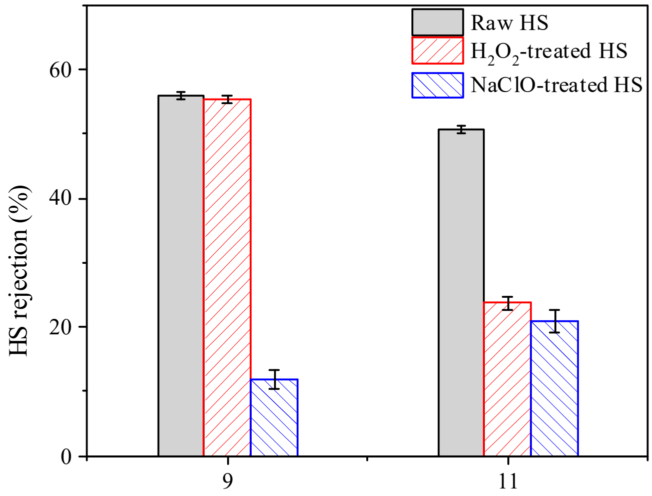

Figure S2. Rejection of HS before and after reacting with H_2O_2 and NaClO. The ratio of oxidant to DOC: 13:1; reaction time: 6 h.

Reference

1. Li, K.; Huang, T.; Qu, F.; Du, X.; Ding, A.; Li, G.; Liang, H. Performance of adsorption pretreatment in mitigating humic acid fouling of ultrafiltration membrane under environmentally relevant ionic conditions. *Desalination* **2016**, *377*, 91–98, doi:10.1016/j.desal.2015.09.016.

2. Hao, Y.; Moriya, A.; Maruyama, T.; Ohmukai, Y.; Matsuyama, H. Effect of metal ions on humic acid fouling of hollow fiber ultrafiltration membrane. *J. Membr. Sci.* **2011**, *376*, 247–253, doi:10.1016/j.memsci.2011.04.035.
3. Katsoufidou, K.S.; Sioutopoulos, D.C.; Yiantsios, S.G.; Karabelas, A.J. UF membrane fouling by mixtures of humic acids and sodium alginate: Fouling mechanisms and reversibility. *Desalination* **2010**, *264*, 220–227, doi:10.1016/j.desal.2010.08.017.
